# High Grade Glioma Treatment in Elderly People: Is It Different Than in Younger Patients? Analysis of Surgical Management Guided by an Intraoperative Multimodal Approach and Its Impact on Clinical Outcome

**DOI:** 10.3389/fonc.2020.631255

**Published:** 2021-02-24

**Authors:** Giuseppe Maria Vincenzo Barbagallo, Roberto Altieri, Marco Garozzo, Massimiliano Maione, Stefania Di Gregorio, Massimiliano Visocchi, Simone Peschillo, Pasquale Dolce, Francesco Certo

**Affiliations:** ^1^ Department of Neurological Surgery, Policlinico "G. Rodolico" University Hospital, Catania, Italy; ^2^ Interdisciplinary Research Center on Brain Tumors Diagnosis and Treatment, University of Catania, Catania, Italy; ^3^ Department of Neuroscience, University of Turin, Turin, Italy; ^4^ Neurosurgical Unit, Catholic University, Rome, Italy; ^5^ Department of Public Health, University of Naples Federico II, Naples, Italy

**Keywords:** elderly, glioblastoma, glioma, 5ALA, geriatric population, brain tumor, ICT, IOUS

## Abstract

**Objective:**

Age is considered a negative prognostic factor for High Grade Gliomas (HGGs) and many neurosurgeons remain skeptical about the benefits of aggressive treatment. New surgical and technological improvements may allow extended safe resection, with lower level of post-operative complications. This opportunity opens the unsolved question about the most appropriate HGG treatment in elderly patients. The aim of this study is to analyze if HGG maximal safe resection guided by an intraoperative multimodal imaging protocol coupled with neuromonitoring is associated with differences in outcome in elderly patients versus younger ones.

**Methods:**

We reviewed 100 patients, 53 (53%) males and 47 (47%) females, with median (IQR) age of 64 (57; 72) years. Eight patients were diagnosed with Anaplastic Astrocytoma (AA), 92 with Glioblastoma (GBM). Surgery was aimed to achieve safe maximal resection. An intraoperative multimodal imaging protocol, including neuronavigation, neurophysiological monitoring, 5-ALA fluorescence, ^11^C MET-PET, navigated i-US system and i-CT, was used, and its impact on EOTR and clinical outcome in elderly patients was analyzed. We divided patients in two groups according to their age: <65 and >65 years, and surgical and clinical results (EOTR, post-operative KPS, OS and PFS) were compared. Yet, to better understand age-related differences, the same patient cohort was also divided into <70 and >70 years and all the above data reanalyzed.

**Results:**

In the first cohort division, we did not found KPS difference over time and survival analysis did not show significant difference between the two groups (p = 0.36 for OS and p = 0.49 for PFS). Same results were obtained increasing the age cut-off for age up to 70 years (p = 0.52 for OS and p = 0.92 for PFS).

**Conclusions:**

Our data demonstrate that there is not statistically significant difference in post-operative EOTR, KPS, OS, and PFS between younger and elderly patients treated with extensive tumor resection aided by a intraoperative multimodal protocol.

## Introduction

Elderly population was defined by the United Nations as people aged >60 years However, the World Health Organization (WHO) set the limit at 65 years; improvement of wellness, health and lifestyle conditions suggests that this limit could be moved up to 70 years, although this is still debated (1). Geriatric population increases rapidly, at a projected 2.9%/year increment by year 2050 (2). Glioblastoma (GBM), the third most frequent tumor of the Central Nervous System (CNS) (14.9%) and the first among malignant ones (47.1%), is usually diagnosed at a median age of 64 years (3). The estimated incidence of GBM in the elderly patients in the United States is 6000/year (4), with rising incidence in patients >70 years in the last decade (5). Predictably, this rate should further increase within few years as life expectancy is continuously growing up. Age is a negative prognostic factor and HGG in elderly patients seem to have a most aggressive behavior because of clinical and genetic features (6–8). Although the population in such age range is constantly increasing, because of the frailty of elderly and the well-known aggressivity of HGG, many neurosurgeons and neuro-oncologists remain skeptical about the benefits of aggressive resective surgery in the geriatric population and brain biopsy or limited cytoreductive surgery are commonly used to obtain histological diagnosis. As a consequence, >65 years HGG patients were not even involved in clinical trials for new treatments (9); indeed, they represent a small group also in the Stupp study on the use of RT combined with temozolomide (TMZ) (10). Innovations in surgical planning and peri-operative medical and anesthesiological support as well as technological advancements provide the opportunity to be more aggressive in the management of CNS tumors, with less post-operative complications (11). And such opportunity opens the unsolved question about the most appropriate treatment in elderly patients.

The aim of this study is to analyze if HGG maximal safe resection guided by an intraoperative multimodal imaging protocol coupled with neuromonitoring is associated with differences in outcome in elderly patients versus younger ones, with regard to post-operative Karnofsky Performance Score (KPS), Overall Survival (OS), the Progression Free Survival (PFS) and Extent of Tumor Resection (EOTR). Specifically our primary outcome is to check if there are difference between the OS of young and elderly population. The secondary outcomes are to check if there are difference between the EOTR, post-operative KPS and PFS of the two groups.

## Materials and Methods

We have retrospectively analyzed all patients surgically treated at University Hospital of Catania from January 2014 to May 2020 and followed up until now. Inclusion criteria were: histopathological diagnosis of HGG; KPS > 60; feasible GTR of enhancing nodule (EN) according to preoperative MRI; age >18 years old; positive 11C-methionine-positron emission tomography (11C-MET-PET).

Exclusion criteria were: low KPS (≤ 60) and poor general conditions; unfeasible GTR due to tumor location (i.e. eloquency) or multifocality, recurrences, adjuvant treatment not performed at our institution, patients loss at follow-up.

In the time period before indicated 117 patients affected by gliomas were admitted at our Neurosurgical Unit. One hundred patients, 53 (53%) males and 47 (47%) females, with median (IQR) age of 64 (57; 72) years meet the inclusion criteria. In all cases postoperative adjuvant therapies included radiotherapy and TMZ (Stupp regimen).

Eight patients were diagnosed with Anaplastic Astrocytoma (AA), 92 with Glioblastoma (GBM) (WHO 2016).

The KPS was used for clinical evaluation and the mean (± standard deviation) preoperative KPS was 75.1 (±13.1). The immediate post-op KPS, as well as KPS at 5 months after surgery, was recorded.

Before and after (within 48 h) surgery all patients underwent MRI with the following sequences: pre- and post-gadolinium T1, T2, T2-FLAIR, DWI, DTI and spectroscopy. Tumor volumes were calculated by neurosurgeons with experience in neuro-oncology and by neuroradiologists (12). The Horos software for MacOs was used for manual segmentation of T1 3D volumetric images. The enhancing nodule (EN) was calculated on pre and post-operative MRI. Necrotic and cystic areas present in the EN were also considered in EN volume. Preoperatively, mean (± standard deviation) maximal tumor diameter was 43.3 (±16.9).

Surgery was always performed aiming to safely remove as much enhancing tumor as possible.

A multimodal intraoperative protocol, including 5-ALA fluorescence, neuronavigation (Medtronic StealthStation™ S7 or S8), neurophysiological monitoring with MEPs, SEPs and cortical-subcortical DES, i-CT (CereTom or BodyTom, Neurologica, US), and navigated bi-dimensional US system (MyLab Twice™ Esaote), was used in all cases to guide safe resection of the tumor. Surgery was stopped in proximity of eloquent areas to avoid post-operative deficits (motor responses at 10 mA stimulation with subcortical DES).

Extent Of Tumor Resection (EOTR) was calculated as preopVol − postopVol/preopVol × 100. We considered as Gross Total Resection (GTR) the complete removal of the EN, as Subtotal Resection (STR) an EOR >75% but less than 99% and as biopsy EOR <75%.

OS was calculated from the date of surgery to date of death, while PFS from date of surgery to date of radiological progression disease (PD) according to the RANO criteria.

Follow-up cut-off for the survival analysis was 2 years after surgery. We divided the entire cohort in two groups according to patients’ age: <65 and >65 years. Clinical (age, sex, and KPS), neuroradiological and histological data were recorded and analyzed. We then compared surgical and clinical results (EOTR, early post-operative and follow-up KPS, OS and PFS) of the above groups.

In order to further investigate possible changes in surgical and clinical outcomes related to age difference, we also divided the same patient cohort into <70 and >70 years and all the above variables were reanalyzed accordingly.

A literature search was performed using the PubMed MEDLINE database. The search term “glioma” was combined with the following: “elderly,” “extent of resection,” “extent of tumor resection,” “neuronavigation,” “intraoperative CT,” “intraoperative ultrasound,” “5-ALA,” “neuromonitoring.”

### Statistical Analysis

Assuming a median OS of 8.6 months in the group of patients with age >65 years old (13) and a median OS of 16 months in the group of patients with age <65 years old (14), a two-sided log-rank test with an overall sample size of 100 subjects (50 in each group) achieved 80% power, at a significance level of α = 5%, to detect a difference between the two groups. Power analysis was performed considering that the study lasted 78 months, from January 2014 to May 2020, of which subject accrual (the entry) occurred uniformly in the first 70 months.

Data were reported as mean (± standard deviation) for continuous variables and as frequencies and percentages (%) for categorical data. To determine demographics and clinic-pathological features differences between the groups created according to the age, x^2^ tests or Student’s t-tests were performed, as appropriate. Kaplan-Meier analysis and log-rank test were used to compare OS and PFS between groups.

We used lme4 to perform a longitudinal linear mixed effects analysis, to test for statistical differences between the groups of patients in terms of the variations from early to delayed post-operative of KPS. As fixed effects, we considered the groups of patients and time. As random effects, we had intercepts for subjects to take into account the non-independence that stems from having three measurements, preoperative KPS, immediate postoperative KPS and KPS 5 months after surgery, by the same subject.

The statistical software R version 3.2.5 (R Core Team, 2018) was used for all statistical analyses. A p-value <0.05 was taken as significance level.

## Results

We divided the entire cohort in two groups according to the age: Group A: patients ≥ 65 and Group B: patients < 65.

In Group A, there were 48 patients, 24 (50%) males and 24 (50%) female, 3 (6.2%) patients were diagnosed with AA and 45 (93.8%) with GBM. In Group B there were 52 patients, 29 (55.8%) males and 23 (44.2%) females, 5 (9.6%) patients with AA and 47 (90.4%) with GBM. GTR was performed in 45 Group A patients (93.8%), and 3 (6.2%) underwent STR; in Group B, we performed 48 (92.3%) GTRs and 4 (7.7%) STRs. The two groups were homogeneous for clinical, surgical and pathological features (**Table 1**).

**Table 1:** Clinical and pathological features of patients stratified for age (<65 years old vs. ≥65 years old).

	Totaln = 100	<65 years oldn = 52	≥65 years old n = 48	p value
Sex Female Male	47(47) 53(53)	23(44.2) 29(55.8)	24(50) 24(50)	0.706
Max tumor diameter	43.3(±16.9)	40.9(± 14.7)	45.8(±18.8)	0.206
Tumor type AA GBM	8(8) 82(92)	5(9.6) 40(90.4)	3(6.2) 45(93.8)	0.717
Type of operation 0 1	7(7) 93(93)	4(7.7) 48(92.3)	3(6.2) 45(93.8)	0.999
Preoperative KPS	75.1(±13.1)	76.5(±14)	73.3(±11.9)	0.231

Data are reported as number of patients (%), mean (± standard deviation), as appropriate. p-values are based on χ^2^ test or Student’s t-test, as appropriate. Note that frequencies over classes do not always sum to the total number of patients, because of some missing values.

**Figure 1:**
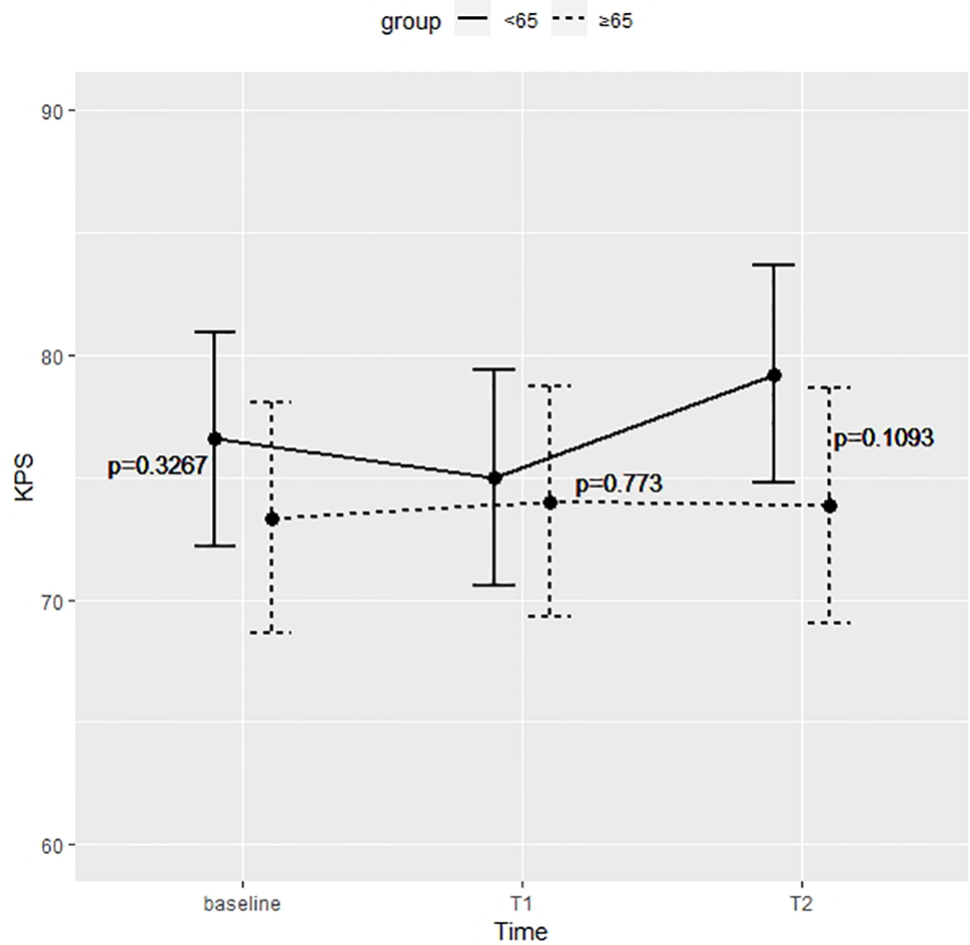
Differences between the two groups were not significant at preoperative time (baseline), immediate postoperative (T1), and 5 months after surgery (T2).

**Figure 1** shows the KPS partial means over time, estimated by mixed effects model. As shown in the figure, differences between the two groups were not significant at each time point.

**Figure 2:**
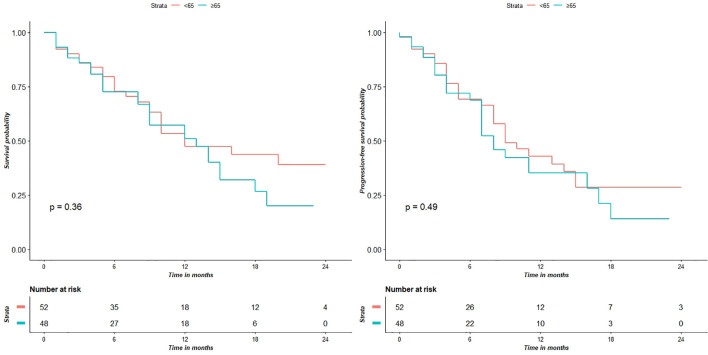
Survival analyses show that the two groups don’t have significantly different for OS (p = 0.36) and PFS (p = 0.49) values.

Survival analysis showed that the two groups did not significantly differed in terms of OS (p = 0.36) and PFS (p = 0.49) (**Figure 2**).

We then divided the same patient’s cohort in two other groups, using 70 years as new cut-off: Group C patients aged ≥ 70 and Group D patients < 70. In Group C there were 32 patients, 16 (50%) males and 16 (50%) females, 2 (6.2%) patients were diagnosed with AA and 30(93.8%) with GBM. Group D included 68 patients, 37 (54.4%) males and 31 (45.6%) females, 6 (8.8%) patients with AA and 62 (91.2%) with GBM. In Group C, 30 (93.8%) patients had tumor GTR and 2 (6.2%) STR; in Group D, 63 (92.6%) patients underwent GTR, and 5 (7.4%) STR.

The two groups were homogeneous for clinical, surgical and pathological data (**Table 2**).

**Table 2:** Clinical and pathological features of patients stratified for age (<70 years old vs. ≥70 years old).

	Totaln = 100	<70 years oldn = 68	≥70 years oldn = 32	p value
Sex Female Male	47(47) 53(53)	31(45.6) 37(54.4)	16(50) 16(50)	0.843
Max tumor diameter	43.3(±16.9)	43.5(± 16.4)	42.9(±18.1)	0.887
Tumor type AA GBM	7(7) 93(92.6)	7(7.4) 63(92.6)	2(6.2) 30(93.8)	0.999
Type of operation 0 1	7(7) 93(92.6)	5(7.4) 63(92.6)	2(6.2) 30(93.8)	0.999
Preoperative KPS	75.1(±13.1)	76(±13.4)	72.8(±12.2)	0.262

Data are reported as number of patients (%) or mean (± standard deviation), as appropriate. p-values are based on χ^2^ test or Student’s t-test, as appropriate. Note that frequencies over classes do not always sum to the total number of patients, because of some missing values.

**Figure 3:**
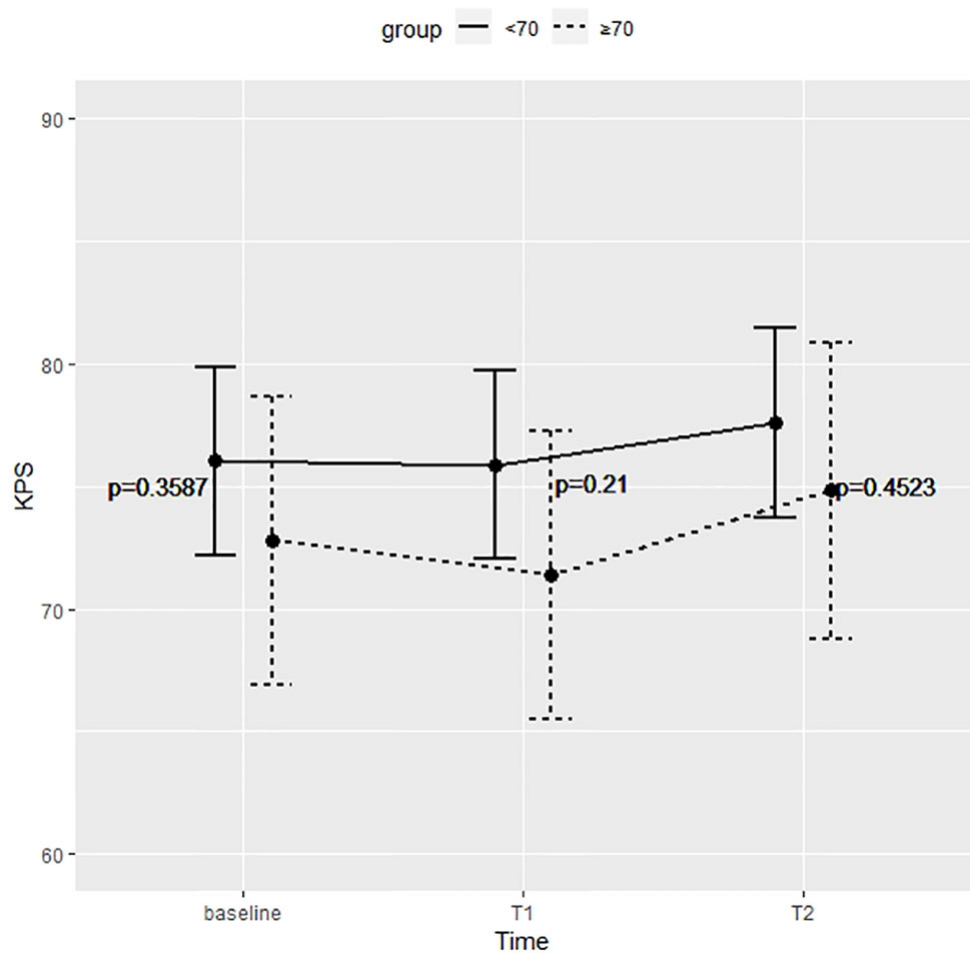
Differences between the two groups were not significant at preoperative time (baseline), immediate postoperative (T1), and 5 months after surgery (T2).

**Figure 4:**
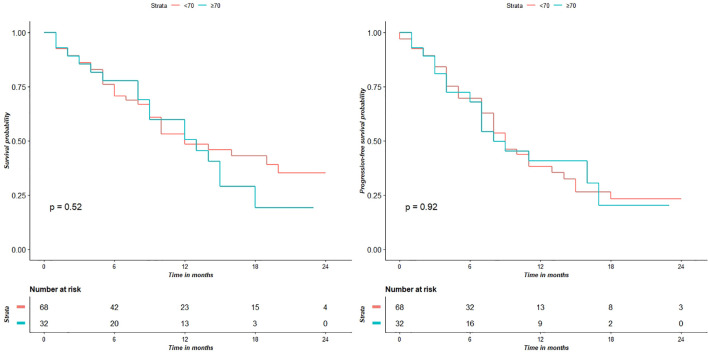
Survival analyses show that the two groups do not have significantly different OS (p = 0.52) and PFS (p = 0.92) values.

**Figure 3** shows the KPS partial means over time, estimated by mixed effects model. Differences between the two groups were not significant at each time point. Survival analysis showed that the two did not significantly differed in terms of OS (p = 0.52) and PFS (p = 0.92) (**Figure 4**).

## Discussion

### “Conservative” Versus “More Aggressive” Treatment

In the past decades, treatment of elderly patients with HGG was usually based on either conservative measures or limited surgery, like biopsy, followed by RT and/or chemotherapy (15).

The main reason for such “minimalist” strategy in over 65 years HGG patients relies on the high rate of comorbidities and on the significant risk of postoperative complications. Moreover, HGGs in elderly population have a larger volume than in younger people (probably due to concurrent brain atrophy causing late symptoms onset) (16), are more aggressive and present more chemo-resistance because of the higher number of genetic mutations (17, 18).

Indeed, Iwamoto et al. published a study based on SEER (Surveillance, Epidemiology and End Results) cancer registry cases treated between 1994 and 2002, and reported that older GBM patients (more than 65 years) constantly received a less aggressive treatment than younger ones, and their OS was reduced from 14.6 months to only 4 months (19).

In 2012, Oszvald et al. compared EOTR in GBM patients, dividing their overall study population in two groups using 65 years as cutoff: no difference in survival rate was found in patients with similar EOTR despite different age groups. The mean PFS in elderly patients improved in the resection group (7.9 months) versus the biopsy one (3.9 months); mean OS increased from biopsy (4 months) to partial (11.4 months) and complete (17.7 months) resection (20). Since Oszvald’s et al. report, other studies supported the concept of as much extended as possible resection (21–24).

However, not every patient over 65 years can be treated with more aggressive (i.e. extensive) surgical management. Trying to evaluate the likely surgical outcome preoperatively, Chaichana et al. identified a panel of preoperative prognostic factors, which are associated with a worse clinical outcome: KPS < 80, Chronic Obstructive Pulmonary Disease (COPD), pre-existing neurological impairment (motor, language and cognitive deficits) and tumor size > 4 cm (25). However, such prognostic factors were reported as negative by different studies (26–30). Oszvald et al. reported worst prognosis in patients with KPS < 80, brain edema, seizures, venous thromboembolism and cognitive dysfunction (20), and Bauchet et al. proposed to use the Charlson Comorbidity Index (CCI), the Mini-Mental State Examination (MMSE) and the comprehensive geriatric assessment (CGA) as decision-making elements to choose the appropriate management in patients >70 years old (31). So far, only KPS (with 80 as cut off) remains the main prognostic factor in multivariate analysis and age does not represents an univocal prognostic value in all studies including over-70 years patients (32–35) .

Despite the above data and concerns, it is currently accepted that clinical outcome may also be influenced by EOTR and, consequently, by different techniques used intraoperatively to increase the extent of a safe surgery.

Our study reports that in elderly patients it is possible to achieve a similar clinical outcome to younger patients, also by applying a multimodal intraoperative imaging protocol, including neuronavigation, 5-ALA fluorescence, i-CT and navigated i-US, coupled with brain mapping, to reach maximal and safe tumor resection.

### Surgical Outcome

The prognostic role of EOTR in HGG is well demonstrated (14, 36, 37). Lacroix et al. showed that 98% resection of the enhancing nodule (EN) in patients suffering from GBM was associated with survival advantage (38). Sanai and Berger, in a retrospective study on 500 patients, challenged the doctrine of all or none, demonstrating that EOTR >78% of EN is related to OS improvement. Moreover, they showed that this applies also to cases in which an even greater resection of the EN is performed (39). Following the constant literature increase supporting the relationship between resection rate of EN and OS, some authors investigated the possibility to obtain a further clinical gain (i.e. survival improvement) with a supratotal resection targeting the FLAIR hyperintense area around the EN; however, results are still controversial (36, 37, 40–44).

The aforementioned studies show the prognostic role of surgery in the general population but in the elderly the potential gain of a more extensive tumor resection should be weighed against its risks. In the Glioma Outcome Project the complication rate after craniotomy is 24.2% (8.1% permanent neurological worsening, 10% regional complications, 9.2% systemic complications, and 1.5% mortality). A retrospective study on 81 patients by De Eulate-Beramendi et al. reports a 17.28% complication rate after surgery (77.8% of patients underwent gross or subtotal resection, 22.2% biopsy only); a higher rate is reported after GTR (22).

The Mayo Clinic retrospective analysis on patients who underwent neuronavigation-assisted GBM resection or biopsy reported complications in 24.8% of patients; in the group who received resection (53/105), 11.3%, 7.6 %, 3.8% had neurological, regional and systemic complications, respectively (45). Moreover, the Mayo Clinic study reports a higher incidence of complications following biopsy rather than tumor resection. The authors’ explanation for such findings focuses on lesion site (eloquent areas or deep locations, thus with a higher effect of even little edematous or hemorrhagic alterations).

In a recent meta-analysis, Almenawer et al. did not found higher rates of morbidity and mortality in older patients undergoing extended tumor resection. Indeed, GTR is reported to relieve neurological deficits and reduce morbidity (21).

### Role of 5-ALA Fluorescence in Guiding Resection

Improvement in achieving GTR comes from 5-ALA fluorescence guidance. 5-ALA helps in distinguishing between tumor and normal brain parenchyma or radiotherapy-induced necrosis; strong 5-ALA fluorescence usually correlates with tumor seen on T1-weighted, gadolinium-enhanced, MRI images; it can also help to recognize tumor tissue in recurrent cases. Moreover, recent studies investigated the correlation between different intensities of 5-ALA and tumor cellularity, highlighting the importance of an extended resection of all fluorescent tissue, when safely feasible, aiming to the so-called “supramarginal resection” [32-34]. The use of 5-ALA is gaining credit also in elderly patients’ surgery. Ewelt et al. reported a series of elderly patients treated with 5-ALA fluorescence: these authors achieved partial and complete resection in 29% and 22% of patients, respectively. Considering the group treated with surgery plus adjuvant chemo- and radiotherapy, the reported OS was 8.6, 13.6, and 7.3 months in total resection vs partial vs biopsy, respectively (13). Yet, Young et al. reported a phase III randomized trial in which 5-ALA fluorescence improved quality and extension of resection in elderly patients in comparison to white light surgery (46).

### Role of Neuromonitoring and Awake Surgery in EOTR

Chaichana et al. reported a 20% rate of new postoperative neurological deficits in series of 129 elderly patients (23% had GBM in eloquent areas) operated using neuronavigation and intraoperative neurophysiological monitoring. The information provided by these tools were useful to detect cortical and subcortical motor functional margins of the resection area, thus reducing the rate of postoperative motor deficits. By applying such strategy, GTR (>99%), NTR (>95%) and STR (80-95%) were reached in 30%, 42%, 28% of patients, respectively (25). Unfortunately, despite neuronavigated tractography and intraoperative neurophysiological monitoring, it is impossible to predict language and neuropsycological outcome with asleep craniotomy (47–49).

Grossman et al. reported their experience on awake craniotomy for brain tumors (gliomas and metastases) in the elderly population. Comparing the results of two patient groups, under and over 65 years of age, respectively, no differences in post-surgical outcome were noted. In particular, in HGG surgery GTR was achieved for young and older patients in 70% and 76% of cases, respectively. Moreover, GTR as opposite to STR showed a gain in survival of 3 months (10.8 vs. 7.8) (50).

### Role of Intraoperative Imaging in Tumor Resection

More recently, the use of advanced intraoperative imaging techniques has been spreading among neurosurgeons; i-CT and i-US represent feasible, fast and reproducible technical tools helping surgeons to accurately perform real-time navigation during brain tumor surgery, to analyze anatomical resection margins and identify query tumor remnants, and to obtain early diagnosis of intra- or perioperative complications (i.e. hemorrhage) (51–53). i-US also helps surgeons to correct for brain shift and to suspect, with high sensibility, the presence of tumor remnants. Yet, the use of specific contrast medium improves vascular and margins visualization, particularly in HGGs (52, 54–57). i-CT is useful in evaluating early complications and specifically identify tumor remnants in the surgical field (49, 51, 58). Yet, i-CT images, which also include brain shift-related changes, can be uploaded into the navigation system and used for a *real-time* navigation, providing more accurate data, which are very useful to pursue extended tumor resection. As already reported in recent papers both i-US and i-CT have limitations related to image interpretation. I-US images are frequently altered by the presence of artifacts and detection and localization of tumor remnants is often limited in presence of large surgical cavity or after hemostatic agents application. Combination of i-US with i-CT may overcome the intrinsic limitations of i-US, as the navigated i-CT may be useful to localize small remnants in hidden portions of surgical field, not clearly identified by ultrasounds [58].

### Role of Multimodal Approach in EOTR

Brain tumor surgery is routinely supported by several intraoperative techniques, such as neuronavigation, i-US, i-CT, i-MRI, fluorescence, and neuromonitoring, which are often used independently. Efficacy of preoperative MRI-based navigation is limited by the brain-shift phenomenon, particularly in cases of large or deep-sited tumors. Intraoperative imaging was introduced also to update neuronavigation data, to reduce brain-shift phenomenon-related pitfalls and to increase overall surgical safety. Nevertheless, each intraoperative imaging modality has intrinsic limitations and technical shortcomings. The possibility to combine them in a multimodal intraoperative imaging protocol could overcome some of these limitations. Combining different intraoperative imaging modalities may increase surgical safety and extent of tumor resection. In particular, i-US seems to be highly sensitive to detect residual tumors, but it may generate false positives due to artifacts. Conversely, i-CT is more specific to localize remnants, as it allows a reliable and more timely (i.e. *real time*) updating of navigation data (59, 60). Finally, neuromonitoring and brain mapping improve the chance to identify brain functional edges in order to achieve a maximal safe resection (61). Such strategy can make a more extensive surgical resection feasible, with lower neurological damage, also in elderly population.

### Role of Age in HGG Surgery

Whenever treating HGG in elderly patients, Neurosurgeons should always consider some factors to choose the most appropriate treatment, including clinical presentation, tumor size and shape as well as overall patient health condition. Indeed, in this specific patient population, all of the above factors may have significant impact on the risk-benefit ratio of surgical management, as these patients often have less physiological reserve, and are predisposed to higher surgical complications rate and delays in recovery (11).

We have shown that the intraoperative multimodal approach is useful to achieve >90% GTR rate in all patient groups and there is no significant OS and PFS difference between younger and elderly patients (both 65 and 70 years as cut off).

## Limitations

This is a retrospective, single center, study with patient enrolled from 2014. The new era of molecular classification start from 2016 and there are not data on tumor molecular markers before that time. Anyway the aim of this paper is to underline the safety and efficacy of a multimodal intraoperative approach demonstrating that an aggressive surgery is technically feasible also in elderly patients.

## Conclusion

Our data show that a more extensive surgery is feasible even in the elderly population. We have demonstrated that there is no statistically significant difference in EOTR, OS, PFS, early post-operative and delayed KPS between younger and elderly patients treated with multimodal intraoperative imaging approach. Although our findings should be confirmed by larger studies, they open the way for further investigations.

## Data Availability Statement

The original contributions presented in the study are included in the article/**Supplementary Material**. Further inquiries can be directed to the corresponding author.

## Ethics Statement

Ethical review and approval was not required for the study on human participants in accordance with the local legislation and institutional requirements. The patients/participants provided their written informed consent to participate in this study.

## Author Contributions

RA with FC has written the paper. GB has supervised and reviewed the manuscript. MG, MM, SD, MV, and SP searched the data. PD has performed the statistical analyses. All authors contributed to the article and approved the submitted version.

## Conflict of Interest

The authors declare that the research was conducted in the absence of any commercial or financial relationships that could be construed as a potential conflict of interest.
